# EHMTI-0222. Habituation of visual evoked potentials in migraine: comparison between blinded and non-blinded analyses

**DOI:** 10.1186/1129-2377-15-S1-E1

**Published:** 2014-09-18

**Authors:** A Ambrosini, J Schoenen, D Magis, F Pierelli, G Coppola

**Affiliations:** 1 Headache Unit, IRCCS Neuromed, Pozzilli, Italy; 2 Headache Research Unit, University of Liège, Liège, Belgium; 3 Dept. Medical-Surgical Sciences and Biotechnologies, University of Rome "La Sapienza" Polo Pontino, Latina, Italy; 4 Neuroophtalmology, G.B Bietti Foundation - IRCCS, Rome, Italy

## Background

In most studies migraineurs have an interictal habituation deficit of visual evoked potentials (VEP) that normalizes during the attack. This, however, was not confirmed by some who suggested that it is due to non-blinded analyses.

## Aim

To compare blinded and non-blinded analyses of raw VEP single-trial signals by two different investigators in healthy volunteers (HV) and migraineurs.

## Methods

Pattern-reversal VEP were recorded in 22 HV , 44 migraineurs without (MO: n=23) or with aura (MA: n=21) and in 24 patients during an attack. Two researchers, one of which was totally blinded to subjects and diagnosis, independently analysed single trials and calculated habituation slopes.

## Results

In both the blinded and the non-blinded analysis VEP habituation was normal in HV (slope -0.12 & -0.2 respectively), but deficient in both migraine groups: MO (+0.02 & +0.09), MA (+0.03 & +0.03). The difference between HV and MO or MA was significant for the blinded (p=0.03 or p=0.001) and the non-blinded procedure–(p=0.03 or p=0.003). Ictal VEP habituation was normal in both blinded and the non-blinded analyses (-0.21 & -0.23; p=0.001 vs. interictal). Intraindividual habituation slope was similar between blinded and non-blinded analyses. Data obtained by non-blinded and the blinded procedure significantly correlated (overall R=0.780. p<0.0000001).

## Conclusion

We confirm in a blinded analysis of raw signals that migraineurs present interictally a significant deficit of VEP habituation, similarly to non-blinded analysis of the same traces. The discrepant results found in some studies can thus not be explained by blinding, but rather by patient-related differences.

No conflict of interest.

